# Supplementary material for: Characterization of Isolated Human Astrocytes from Aging Brain
Source: Int J Mol Sci. 2025 Apr 5;26(7):3416. doi: 10.3390/ijms26073416 (PMC11990013; doi:10.3390/ijms26073416)
Supplement: Supplementary file 1 [file ijms-26-03416-s001.zip › Supplemental Figure S1.pdf]

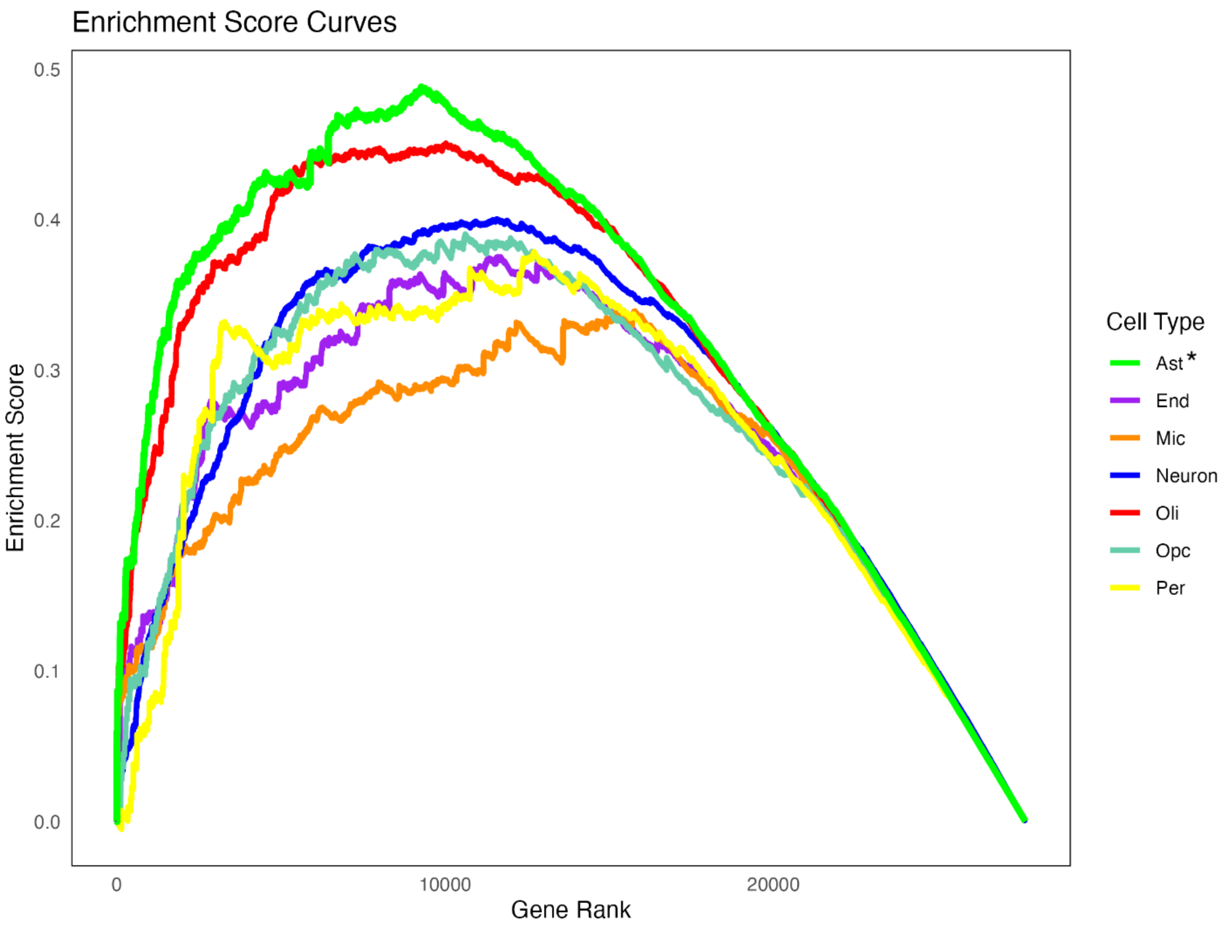

Supplemental Figure S1. Cell-Specific Gene Set Enrichment Analysis. This fGSEA plot illustrates the enrichment score (y-axis) for various cell types along gene ranks (x-axis) using a list of 5,641 cell-specific genes and highlighting the differences in pathway enrichment across the ranked gene list. The curve for astrocytes (Ast; green) shows their significant enrichment (*p-value < 0.05), while other cell types, shown in different colors, do not reach statistical significance.
